# An AI-based approach for modeling the synergy between radiotherapy and immunotherapy

**DOI:** 10.1038/s41598-024-58684-6

**Published:** 2024-04-08

**Authors:** Hao Peng, Casey Moore, Yuanyuan Zhang, Debabrata Saha, Steve Jiang, Robert Timmerman

**Affiliations:** 1 Departments of Radiation Oncology, University of Texas Southwestern Medical Center, Dallas, TX USA; 2 Medical Artificial Intelligence and Automation Laboratory, University of Texas Southwestern Medical Center, Dallas, TX USA; 3 Departments of Immunology, University of Texas Southwestern Medical Center, Dallas, TX USA

**Keywords:** Radiotherapy, Cancer immunotherapy

## Abstract

Personalized, ultra-fractionated stereotactic adaptive radiotherapy (PULSAR) is designed to administer tumoricidal doses in a pulsed mode with extended intervals, spanning weeks or months. This approach leverages longer intervals to adapt the treatment plan based on tumor changes and enhance immune-modulated effects. In this investigation, we seek to elucidate the potential synergy between combined PULSAR and PD-L1 blockade immunotherapy using experimental data from a Lewis Lung Carcinoma (LLC) syngeneic murine cancer model. Employing a long short-term memory (LSTM) recurrent neural network (RNN) model, we simulated the treatment response by treating irradiation and anti-PD-L1 as external stimuli occurring in a temporal sequence. Our findings demonstrate that: (1) The model can simulate tumor growth by integrating various parameters such as timing and dose, and (2) The model provides mechanistic interpretations of a “causal relationship” in combined treatment, offering a completely novel perspective. The model can be utilized for in-silico modeling, facilitating exploration of innovative treatment combinations to optimize therapeutic outcomes. Advanced modeling techniques, coupled with additional efforts in biomarker identification, may deepen our understanding of the biological mechanisms underlying the combined treatment.

## Introduction

Personalized ultra-fractionated stereotactic adaptive radiotherapy (PULSAR) aims to deliver tumoricidal doses in a pulse mode with long intervals. It offers several advantages. Longer intervals (weeks or months) allow more normal tissue recovery after injury. At the same time, longer interval provides time for the tumor and tumor microenvironment (TME) to undergo changes, and thus allows for more meaningful adaptation based upon the evolving characteristics of the tumor (e.g., the change of tumor size, shape, and biomarker expression). Achieving this in conventional radiation therapy is challenging because not only is the tumor unlikely to undergo significant changes between two closely spaced radiation doses, but repeated dosing over a short interval may impede potential synergies in combined radiation therapy and immunotherapy. Despite promising progress, combination of the two treatments has yielded lackluster results in both clinical and preclinical studies. Whether synergy exists or if efficacy resides within each modality, remains an intriguing research question.

One aspect of interest is determining the optimal timing and sequence to harness the immune-mediated antitumor effects in combined radiation therapy and immune checkpoint blockade (ICI) therapy (e.g., PD-L1, CTLA4)^1–10^. For instance, one clinical trial studied the outcome of multisite Stereotactic Body Radiation Therapy (SBRT) followed by pembrolizumab, reporting an overall response rate of 13.2% in advanced solid tumors^6^. The PEMBRO-RT phase 2 clinical trial in non-small cell lung cancer demonstrated a doubling in overall response when patients were treated with SBRT (3 × 8 Gy) followed by pembrolizumab^7^. The synergistic impact has been extensively explored in preclinical models as well. One study delivered 10–24 Gy in 1–3 daily, or every other day fractions and began PD1/PD-L1 checkpoint blockade therapy within a day of radiation^5^. Another preclinical study included 3 daily SBRT fractions of 8 Gy, followed by anti-CTLA4 treatment, beginning on the day of the last fraction^8^. Both studies demonstrated additive benefits when ICI blockade is given concomitantly with radiation. Moreover, there are conflicting reports regarding the optimal timing of PD-L1 therapy in relation to radiation. While some studies showed no additive benefit when PD-L1 therapy was administered 6 days after a single dose of radiation^9^, others suggested clear benefits of PD-L1 therapy given even 21 days after radiation^10^.

Our team has explored PULSAR with anti-PD-L1 therapy using immune activated and resistant syngeneic mouse models^11^. More details about the experimental design can be found in Moore et al^11^. Tumor growth was studied as a function of radiation dose and scheduling. The results revealed that the effectiveness of anti-PD-L1 therapy depended on how the radiation was sequenced relative to the timing of ICI therapy. For instance, less tumor control was seen with the anti-PD-L1 drug if radiotherapy was given daily or every 4 days, compared to an interval of 10 days. This motivates us to dive deeper into answering the following question: How do the responses resulting from radiation therapy and ICI therapy interact temporally with each other? The Lewis Lung Carcinoma (LLC) cell line was chosen as the tumor model due to its aggressive nature and immunogenic properties. Acknowledged as a “cold” tumor characterized by low T cell infiltration and high myeloid suppressive cell infiltration, LLC offers valuable insights into the temporal behavior of the adaptive immune response^12^. This is crucial for studying synergy since it requires longer time for the adaptive immune response to develop and reach its full effectiveness. In addition, differing from most preclinical models using radiation and with either daily or every other day fractions^5,8–10^, we tested radiation pulses with a longer spacing.

**Figure 1:**
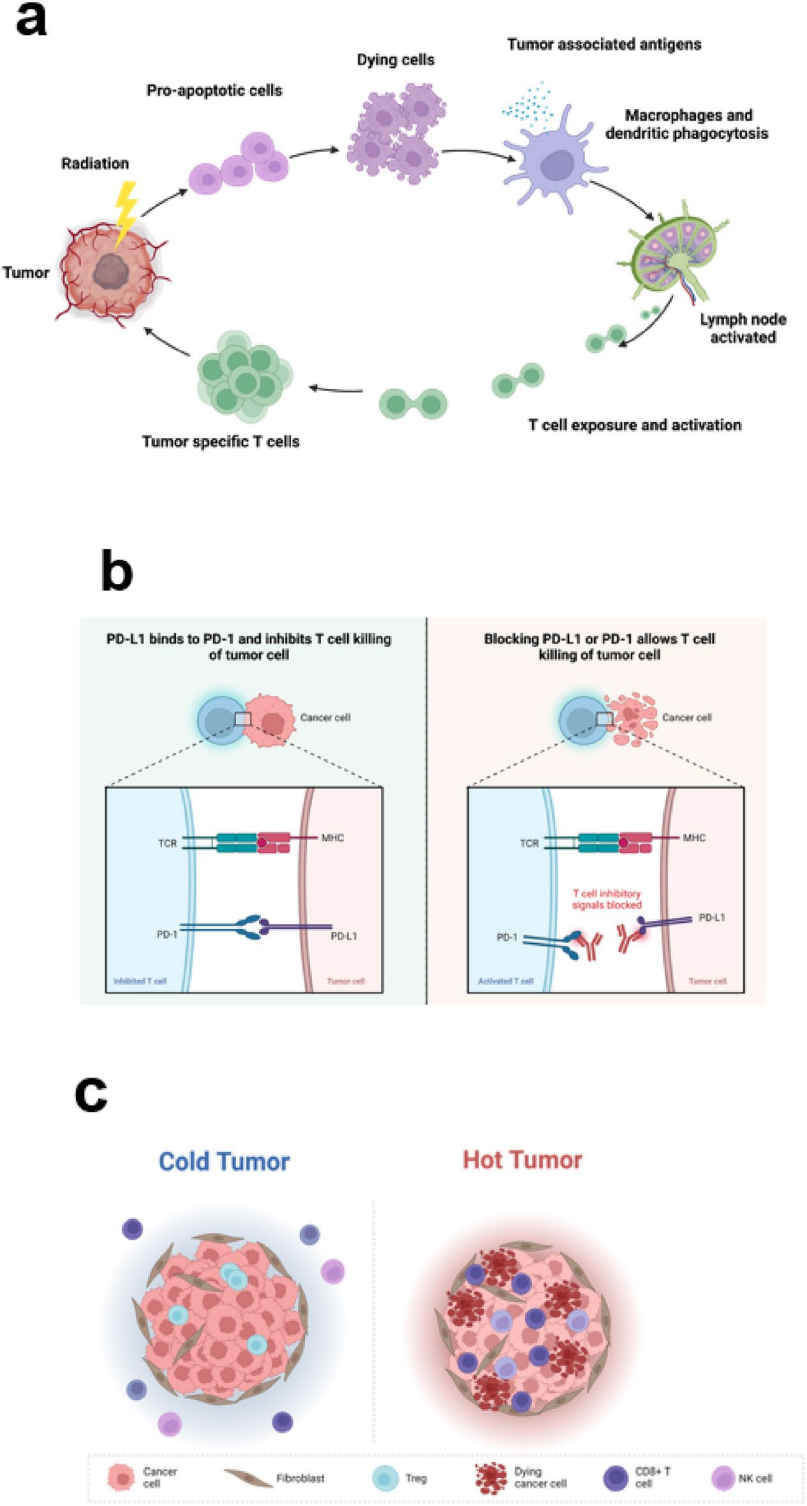
Illustration of three key processes involved in combined radio-immunotherapy. (**a**) Radiation kills tumor cells to slow down tumor growth, while simultaneously enacts the exposure of tumor-associated antigens to recruit CD8 + T cells to the tumor site. (**b**) Anti-PD-L1 blocks the binding between PD- and PD-L1, reinvigorating immune response against tumor cells. (**c**) Radiation stimulates the infiltration of T-cells and changes TME.

Our goal is to develop a modeling tool to better understand the interaction between radiation therapy and anti-PD-L1 therapy, discerning whether synergy exists or if efficacy is just confined to each treatment modality independently. Given the complexities of both physical and biological processes involved, it is a challenging task to model the response of individual components such as tumor growth, radiation damage, immune response, TME and T-cell dynamics (Fig. 1). To better understand this, several relevant physical and biological processes are outlined below. For example, tumor growth (including repopulation), without the influence of any treatment adheres to a time-dependent exponential model. The impact of a single radiation pulse can be represented using a linear quadratic (LQ) model^13^. The interplay between radiation and checkpoint inhibitors, like the PD-1 and PD-L1 checkpoint blockade, introduces additional complexity. On one hand, radiation fosters local and systemic immune responses, recruiting CD8 + cytotoxic T cells to tumor sites, through pathways like the c-GAS-STING cytosolic DNA-sensing pathway^14^. This process is time-dependent and lasts several days. On the other hand, radiation-induced T_reg_ cells release interferon-gamma, causing an increase in PD-L1 expression on tumor cells and triggering immune escape within a few days^15–18^. The administration of anti-PD-L1 antibody acts to counteract this process. Simultaneously, T_reg_ cells interact with effector T cells, restraining their functions by secreting inhibitory cytokines like IL-10 and TGF-β. As a result, the net tumoricidal effect in combined radiation therapy and ICI therapy hinges on factors like radiation dose, anti-PD-L1 dose, and relative timing. Furthermore, the modeling of system response becomes complicated due to the dynamics of T cells: (1) The continuous migration of T cells to tumor sites is affected by radiation pulses, and (2) there are variations in radiosensitivity between tumor-resident T cells and newly arrived T cells^19^.

**Figure 2:**
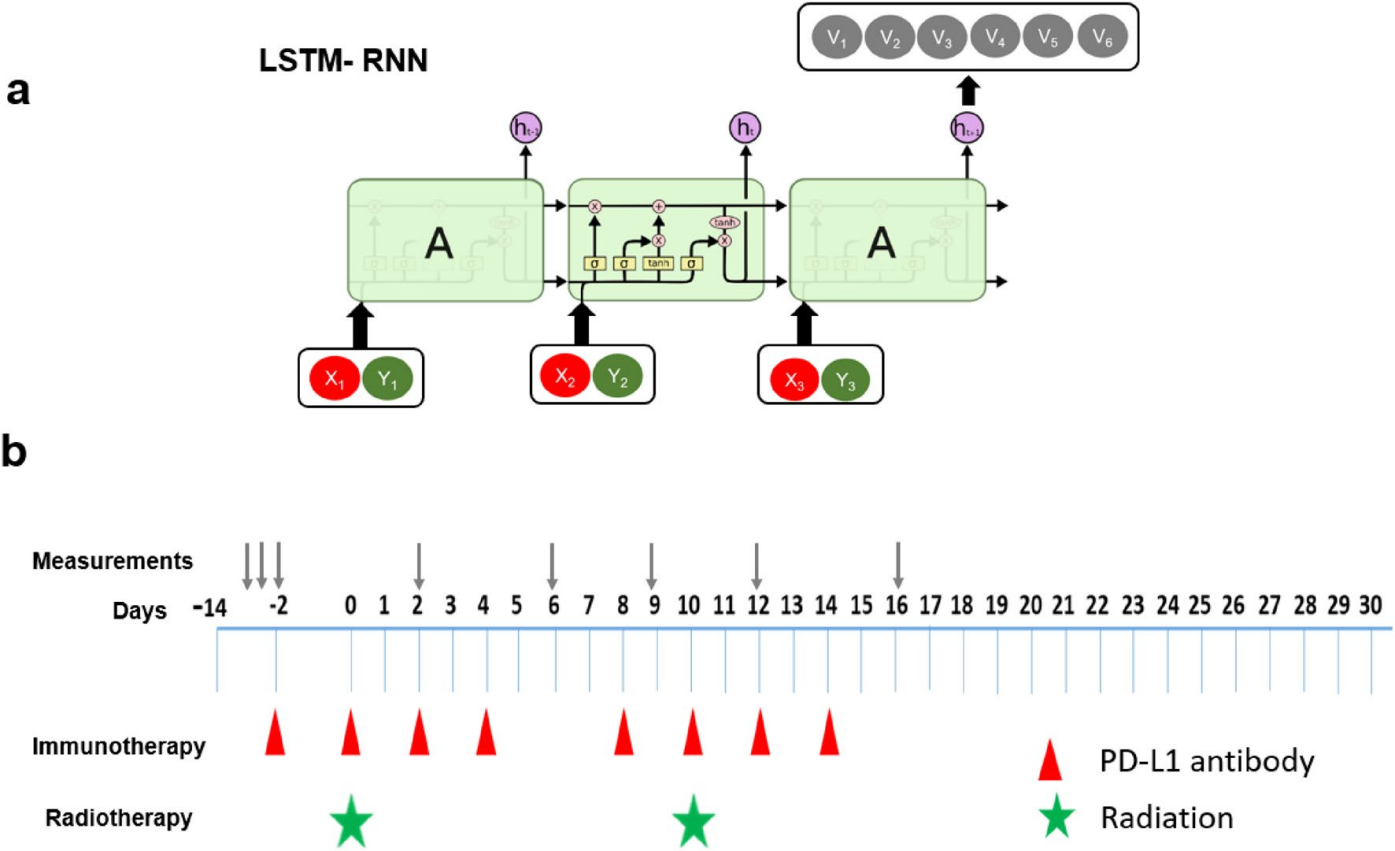
(**a**) The LSTM-RNN model specializes in capturing sequential information. Besides three gates (input, forget, and output), it comprises two internal states: memory cell states (e.g. keeping/discarding formation) and hidden states. *X* represents the radiation input and *Y* represents the anti-PD-L1 input, respectively. (**b**) The timing diagram of the combined therapy in our study. For immunotherapy, either anti-PD-L1 or isotype control was administered. For radiation, different doses were delivered (10, 15, 20, 40 Gy). The first pulse of radiation was always delivered 14 days after the implantation. Tumor volume measurements were carried out sequentially on certain days.

Instead of going with a conventional analytical approach (e.g., solving differential equations), we deployed an AI-based approach using long short-term memory (LSTM) recurrent neural network (LSTM-RNN)^20^. RNN is a generalization of feed-forward neural networks that have an internal memory, offering unique strengths in identifying correlation in sequential signals^21–23^. Radiation and anti-PD-L1 can be viewed as two external stimulations occurring in a temporal sequence (Fig. 2a). It has the potential to allow us to disentangle the interplay between the two treatments, offering some mechanistic interpretations regarding a “causal relationship” from a novel perspective.

## Materials and methods

### Small animal experiments

The experimental protocols in this manuscript were approved by the Institutional Animal Care and Use Committee (IACUC) at UT Southwestern. We confirm that all animal procedures were performed in accordance with the animal experimental guidelines set by the IACUC at UT Southwestern. UT Southwestern uses the “Guide for the Care and Use of Laboratory Animals” when establishing animal research standards. We also confirm that the study was reported in accordance with ARRIVE guidelines (https://arriveguidelines.org). Different timing and dose were studied as illustrated in Fig. 2b and more details can be found in Table S1 in Supplemental materials. Female C57BL/6 J mice were purchased from Charles River or Jackson Laboratories at six to eight weeks old. LLC was derived from lung cancer of the C57BL/6 line. Tumor cells were injected subcutaneously on the right leg of mice. Either 200 μg anti-PD-L1 or 200 μg isotype control was administered i.p. to mice every 2 days. Local irradiations were conducted on a dedicated x-ray irradiator (X-RAD 32, Precision X-ray, Inc.) (see Figure S1 in Supplemental Materials). Tumor-bearing mice were anesthetized using isoflurane and mounted on an acrylic bed equipped with a nose cone. Various collimator sizes were developed to form the field of view, which depended on tumor size. The mouse was positioned such that the source-to-tumor surface distance was 20 cm, and the tumor was in the center of the x-ray beam. The energy of the x-ray was set to 250 kVp and current was set to 15 mA for irradiation. The dose rate under this condition was 19.468 Gy/min, which was calibrated using a PTW 31,010 ionization chamber and a PTW UnidosEelectrometer (PTW North America Corporation, New York, NY) in accordance with the AAPM TG-61 protocol. The mice were randomized to treatment groups when tumors reached 150–200 mm^3^ and tumors were treated with either anti-PD-L1 or isotype control. The tumor volumes were measured manually by length (x), width (y), and height (z) and calculated as tumor volume = xyz/2. If each of length, width or height of tumor is larger than 2 cm, the tumor volume is larger than 1500 mm^3^, or the mouse had significant ulceration in the tumor (see Fig. S1 in Supplemental materials), the mouse reached the survival endpoint and was euthanized by exposure to CO_2_.

### Data pre-processing

For each treatment group, seven or eight animals were studied. Measuring the volume at the end of each treatment cycle became progressively challenging, highlighting a limitation in our current study. From a machine learning perspective, significant uncertainty in volume measurements diminishes the discernible correlation, if any, between an input sequence and an output sequence. Data augmentation was thus necessary for training the LSTM-RNN model. The details of data processing are summarized below. The total time course was set to be 18 days for all groups to ensure data consistency. Although for some animals, the measurement was performed up to 40 days, an increased percentage of animals had missing data towards the survival endpoint due to severe ulceration.

**Figure 3:**
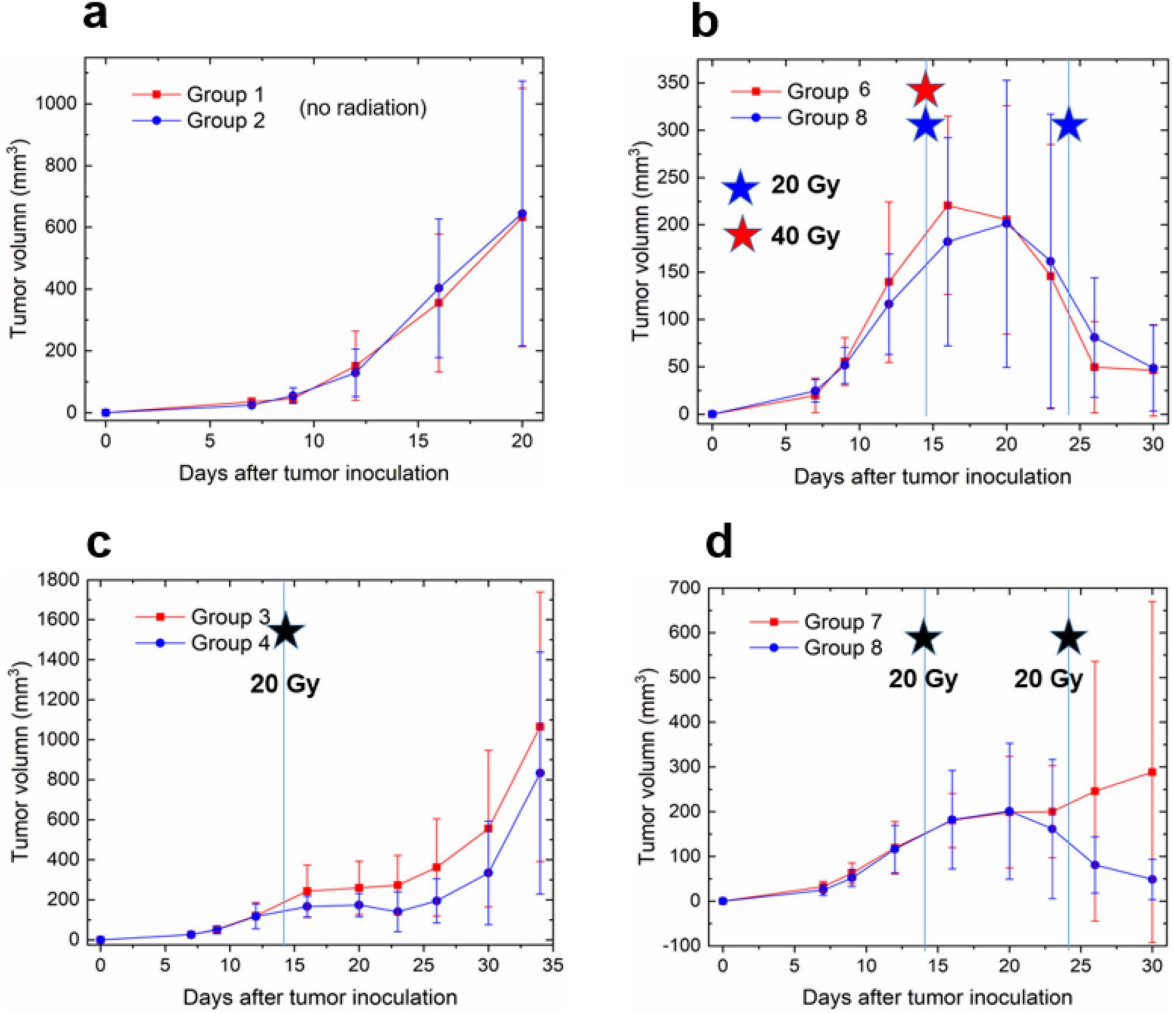
Illustration of tumor volume measured under different delivery schemes according to Fig. 2b. The vertical line on day 14 represents the first radiation pulse. (**a**) Group 1 (isotype control) and group 2 (PD-L1 antibody, every other day), both without radiation applied. (**b**) Group 6 (a single pulse of 40 Gy) and group 8 (two pulses of 20 Gy separated by 10 days), both with PD-L1 antibody administered every other day. (**c**) Group 3 (control) and group 4 (PD-L1 antibody, every other day), both with a single pulse of 20 Gy delivered on day 14. (**d**). Group 7 (control) and group 8 (PD-L1, every other day), both with two pulses delivered with an interval of 10 days.

**Figure 4:**
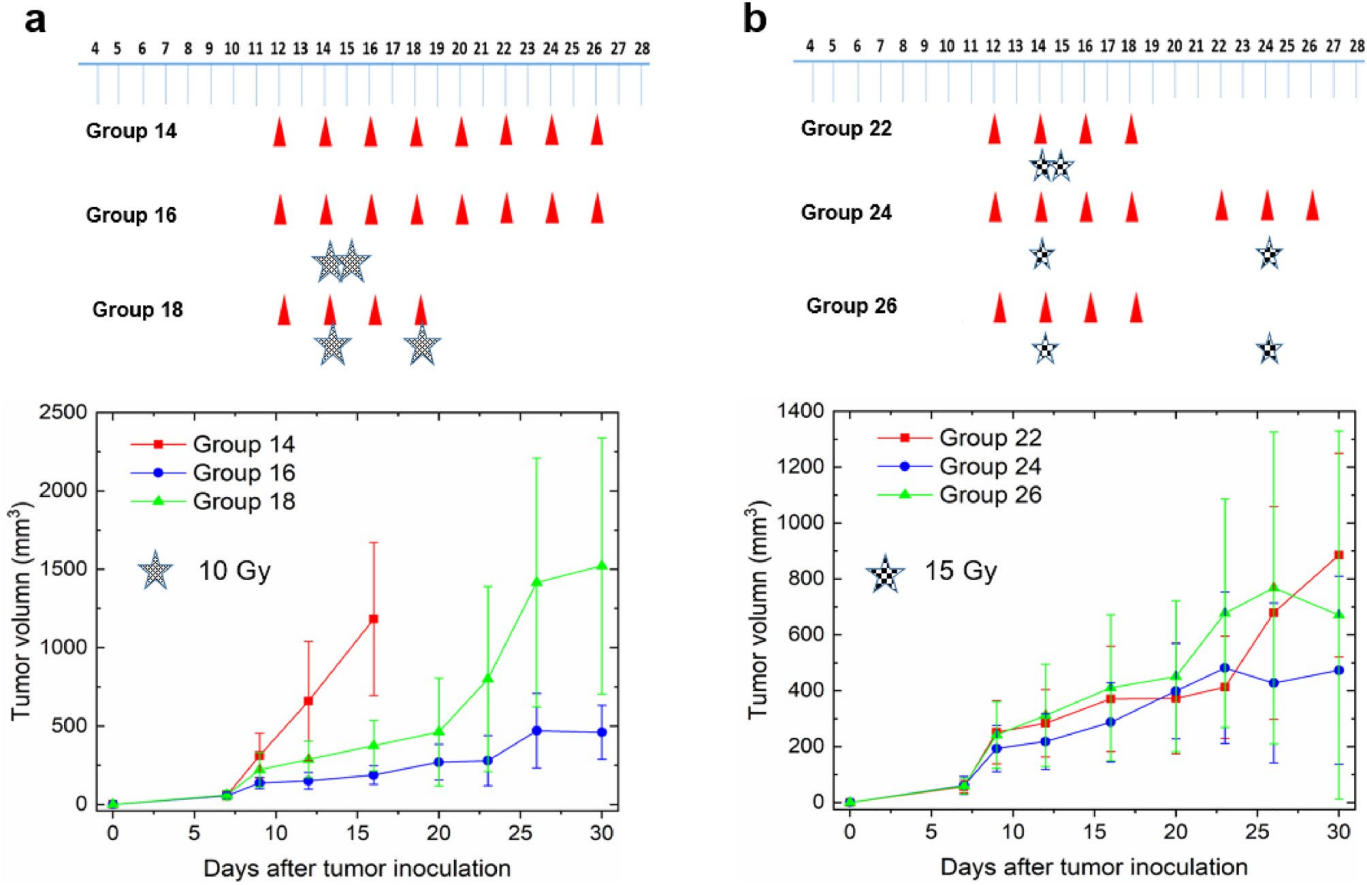
Illustration of tumor volume measured change under multiple delivery schemes, for a total dose of 20 Gy (groups 16 and 18) or 30 Gy (groups 24 and 26) (note that different Y-axis scales are used). The first pulse of radiation was delivered 14 days after tumor inoculation. Different from Fig. 3, PD-L1 antibody was not administered after the first pulse in some groups.

We had 26 treatment groups in total, with the details of each group summarized in the Supplemental materials. We initially computed the mean and standard deviation using the tumor volumes of seven or eight animals in each group on a specific measurement day. Assuming a Gaussian distribution for the tumor volume on a given day, we generated 50 samples randomly from the distribution. To address the issue of large error bars in measured volume (Figs. 3 and 4), we intentionally reduced the standard deviation by a factor of five, which we believe is achievable by employing more accurate volume measuring techniques such as cone beam computed tomography (CBCT) or positron emission tomography (PET). We recognize that the data augmentation method outlined above may lack statistical rigor, but it is the only feasible approach given the available data. As a supplementary point, the incorporation of 50 samples through data augmentation can also be viewed as a strategy to alleviate overfitting and improve generalization.

1300 samples were used (26 groups and 50 samples per group). Each sample consisted of a sequence of 18 steps (days) with two inputs and one output. The two inputs corresponded to radiation dose and anti-PD-L1, respectively. For radiation, a single value encoded the dose, representing the killing effect using the LQ model which is dose dependent. Otherwise, it was set to zero. In the case of anti-PD-L1, the input was coded as 1 (isotype control), 2 (anti-PD-L1), and 0 if no dosage. These two inputs were concatenated at each time step. The output represented the change in tumor volume between two consecutive measurements. For example, if we conducted six measurements for an animal, there would be five non-zero values. Positive change signifies volume increase, while negative change signifies volume decrease. Such a choice is due to the design of our animal experiments preceding the AI modeling. A more continuous measurement approach in the future will address this limitation.

### RNN modeling and training details

We developed the LSTM-RNN model using the Pytorch package. The model consisted of 100 hidden units at each time step and a fully connected layer in the last time step to predict volume change. In other words, the model used a “many to one” rather than a “many to many” structure. The ADAM algorithm with the following settings was used for optimization (learning rate = 0.001, beta1 = 0.9, beta2 = 0.999, epsilon = 1 × 10^−8^)^24^. Training was implemented on a PC with a NVIDIA GeForce RTX 3090 and an Intel(R) Core(TM) i7-10700 K CPU. The batch size was 100 and the total epoch was 5000. The loss functions defined in Eqs. (1) and (2) were calculated based on the non-zero points (e.g. with measurements performed) only. They represent the discrepancy between a predicted volume change ($\Delta {VC{\prime}}_{i}$) and its experimental counterpart ($\Delta {VC}_{i}$), where *i* is the index of measurement and ΔVC may assume either positive or negative values.

1 $${\Delta }VC_{i} = Volume_{i + 1} - Volume_{i} \;\;\left( {{\text{i}} = {1},{ 2},{ 3},{ 4},{ 5}} \right)$$

2 $$Loss = \mathop \sum \limits_{i} \left( {{\Delta }VC_{i} - {\Delta }VC^{\prime}_{i} } \right)^{2}$$

A total of 1200 samples (24 groups with 50 samples each) were utilized, with 80% allocated for training and 20% for testing (100 samples). The cross-validation process involved randomly selecting two groups, excluding them from the training set, and repeating this procedure five times to train five models. These models were then applied to a new sequence to assess their robustness. Notably, this differs from holdout validation as no measurement data were available for the new sequence. In our experiment, slight variations were noted among the five models, and only one was thus selected for further analyses (see Fig. S2 in Supplemental materials). It is important to emphasize that our primary focus does not lie in quantitative aspects, given the inherent challenges in achieving both precision and accuracy.

### Interpretation of hidden units and predictive modeling

To gain deeper insights into the internal signal flow within the LSTM-RNN model, specifically regarding the “causal relationship”, we extract the values of 100 hidden units for all 18 time steps. By performing an arithmetic operation on the maps of hidden units between two groups (e.g., with or without anti-PD-L1, with or without radiation), we attempted to separate the respective effects of each treatment and provide plausible explanations. Additionally, we proceeded to assess the model’s predictive power and estimate the outcomes of new delivery sequences not included in the experiments. To determine overall tumor control, analogous to the endpoint in clinical studies, we utilized the accumulated volume change (AVC) as the criterion (Eq. 3). We tested three cases: case 1 (two pulses of 10 Gy with a 10-day interval), case 2 (two pulses of 20 Gy with a 10-day interval), and case 3 (a single pulse of 40 Gy). For each case, a cycle of anti-PD-L1 on four or two consecutive days was applied. We analyzed AVCs at various offsets ranging between 1 and 14 days. For example, an offset of 5 days implies that 5 days after the delivery of the first radiation pulse, anti-PD-L1 is administered on four consecutive days. While there are numerous permutations to explore, we provided only two cases above to exemplify the potential role of using the LSTM-RNN as an in-silico modeling tool.

3 $$AVC = \mathop \sum \limits_{i = 1}^{5} {\Delta }VC^{\prime}_{i}$$

## Results

### Experimental study

To illustrate the influence of radiation schedules on tumor control in combined treatment, we summarize the experimental results of a subset of groups in Figs. 3 and 4.

In the absence of radiation, there is no significant difference observed between group 1 (without anti-PD-L1) and group 2 (anti-PD-L1) (Fig. 3a). When comparing group 6 (a single pulse of 40 Gy, anti-PD-L1) and group 8 (two pulses of 20 Gy with a 10-day interval, anti-PD-L1) (Fig. 3b), both suppress tumor growth effectively. The tumor control between 40 and 20 Gy appears comparable in the presence of large error bars, as indicated by the data points following the first pulse on day 14. In Fig. 3c where a single pulse of 20 Gy was delivered on day 14, there is a noticeable benefit in tumor control for group 4 (anti-PD-L1) compared to group 3 (without anti-PD-L1), which becomes apparent 1–2 days after the first pulse. In Fig. 3d, when two radiation pulses (20 Gy with a 10-day interval) are delivered, we observe a general trend like Fig. 3c. The difference between group 7 (without anti-PD-L1) and group 8 (with anti-PD-L1), becomes more pronounced after the second radiation pulse.

Figure 4 presents two additional comparisons, including more variations in delivery parameters. In Fig. 4a, rapid tumor growth is observed when only anti-PD-L1 is present (group 14, no radiation). Group 16 (two 10 Gy pulses, separated by 1 day) exhibits improved tumor control compared to group 18 (~ 1500 mm^3^ vs. ~ 500 mm^3^ at day 30). This could be attributed to several factors: (1) a lower survival fraction when a single fraction (or pulse) is delivered compared to two fractions 4 days apart, (2) potential inhibitory impact on the immune response by the second pulse which kills immune cells, and (3) the absence of anti-PD-L1 application after the second radiation pulse. In Fig. 4b, following the first radiation pulse, distinguishing the differences among three groups becomes challenging due to error bars. Despite that, when the second pulse is administered concurrently with anti-PD-L1 (group 24), improved tumor control is achieved compared to the other two groups. The absence of either radiation (group 22, (two 15 Gy pulses, separated by 1 day) or anti-PD-L1 (group 26) comprises the therapeutic effect. Furthermore, when examining group 16 in Fig. 4a and group 22 in Fig. 4b, the sharp decrease evident in Fig. 3b (40 Gy) is not observed, attributable to the reduced total dose.

### Model training and prediction

**Figure 5:**
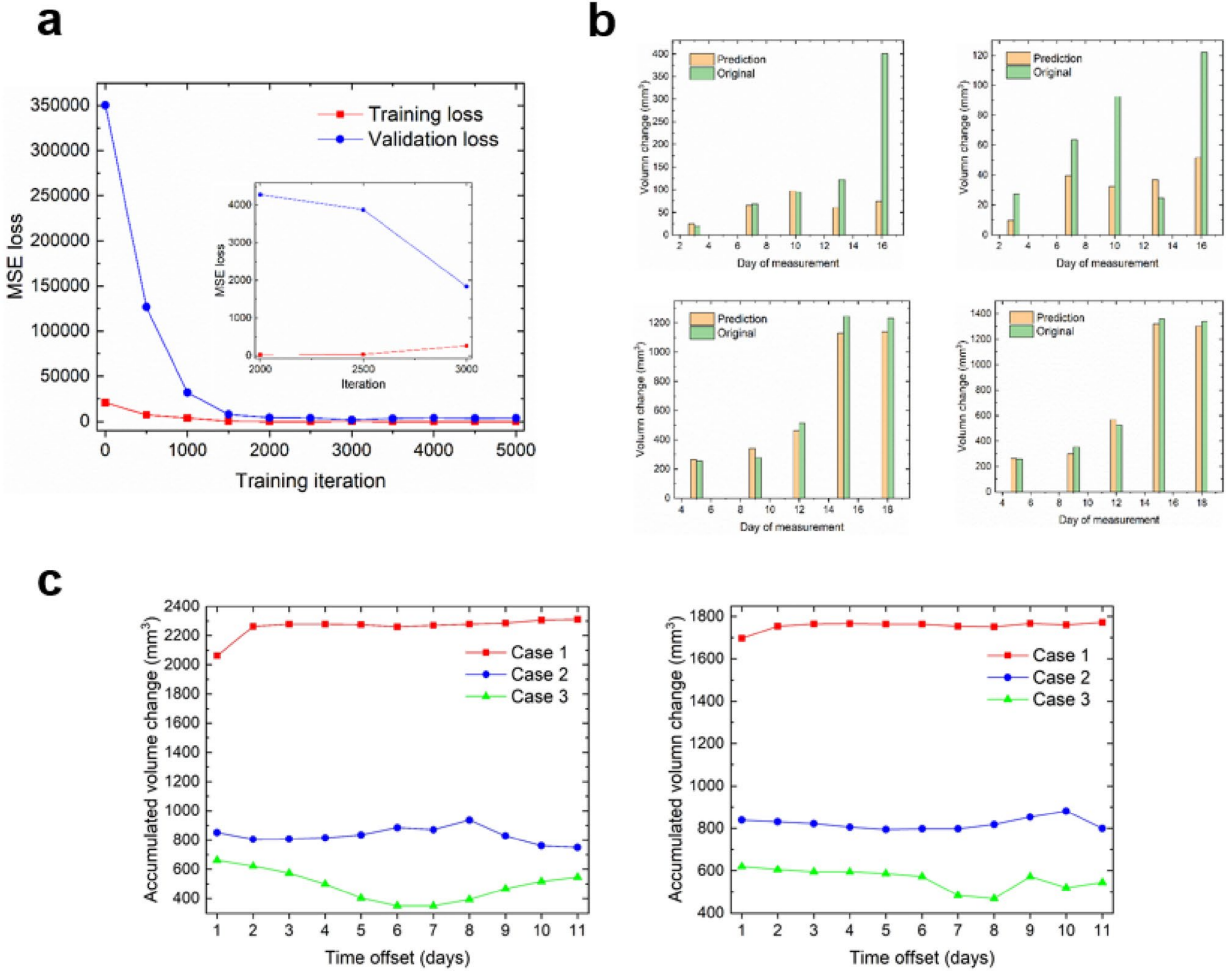
(**a**) Training-loss and validation-loss curves in terms of mean squared error as a function of iteration. Details are shown in the zoom-in figure inset. (**b**) The results of validation for two cases, each case having two groups not included for training (top: groups 12 and 13, bottom: groups 11 and 14). The “original” volume change corresponds to the mean result of 50 samples within each group (after data augmentation). (**c**) The accumulated volume change (AVC) for new delivery schemes not included in the experimental study: case 1 (two pulses of 10 Gy, an interval of 10 days), case 2 (two pulses of 20 Gy, an interval of 10 days), and case 3 (a single pulse of 40 Gy). For all three cases, PD-L1 antibody was administered on either four (left panel) or two consecutive days (right panel), starting at different time offsets (in days) relative to the first radiation pulse.

Figure 5a illustrates the convergence of both the training and validation loss of the LSTM-RNN model after approximately 2000 iterations. The validation loss exhibits a minor deviation from the training loss, suggesting the absence of significant overfitting. The results of the two validation tasks are displayed in Fig. 5b, displaying an overall favorable prediction accuracy. The majority of differences at each time point reside within the range of − 100 to 100 mm^3^, except for a single point in group 11, which surpasses 300 mm^3^ on day 16. Considering that the tumor volume in the control group exceeds 600 mm^3^ around day 20 (Fig. 3a), the predictive accuracy offered by the LSTM-RNN model is deemed satisfactory. The results of other groups are presented in Figure S3 within the supplemental materials.

To illustrate the use of the LSTM-RNN model for in-silico analysis, the response of AVC for new treatment schemes is depicted in Fig. 5c. These schemes involve either two or four consecutive daily administrations of anti-PD-L1, slightly different from every other day administration in the experiments. In the case of a single pulse of 40 Gy (case 3), optimal tumor control is achieved with an offset of 7–8 days. Four doses of anti-PD-L1 result in slightly improved tumor control compared to two doses, with a minimum volume of 350 mm^3^ (left) versus 468 mm^3^ (right). By contrast, for both case 1 and case 2 (two pulses of either 10 Gy or 20 Gy with a 10-day interval), AVC remains relatively stable, except for an abrupt increase observed in case 1 on the left end. These patterns suggest that for case 1 and case 2, the overall synergistic therapeutic effect is anticipated to be similar, irrespective of when anti-PD-L1 is administered after the first radiation pulse. Another observation in case 1 is the noticeable difference in AVC between two and four anti-PD-L1 administrations, with tumor volumes of approximately 1750 mm^3^ (right panel) and 2250 mm^3^ (left panel), respectively. In other words, for a radiation pulse of relatively low dose, such as 10 Gy, doubling the dose of anti-PD-L1 adversely affects the treatment outcome, which is possibly due to treatment toxicities. Nevertheless, these patterns await further validation. They may unveil the authentic interplay between radiation and the immune response, or alternatively, highlight the limited generalization capability of the LSTM-RNN model stemming from the limited number of groups.

### Analysis of hidden units and interpretation of “causal relationship”

**Figure 6:**
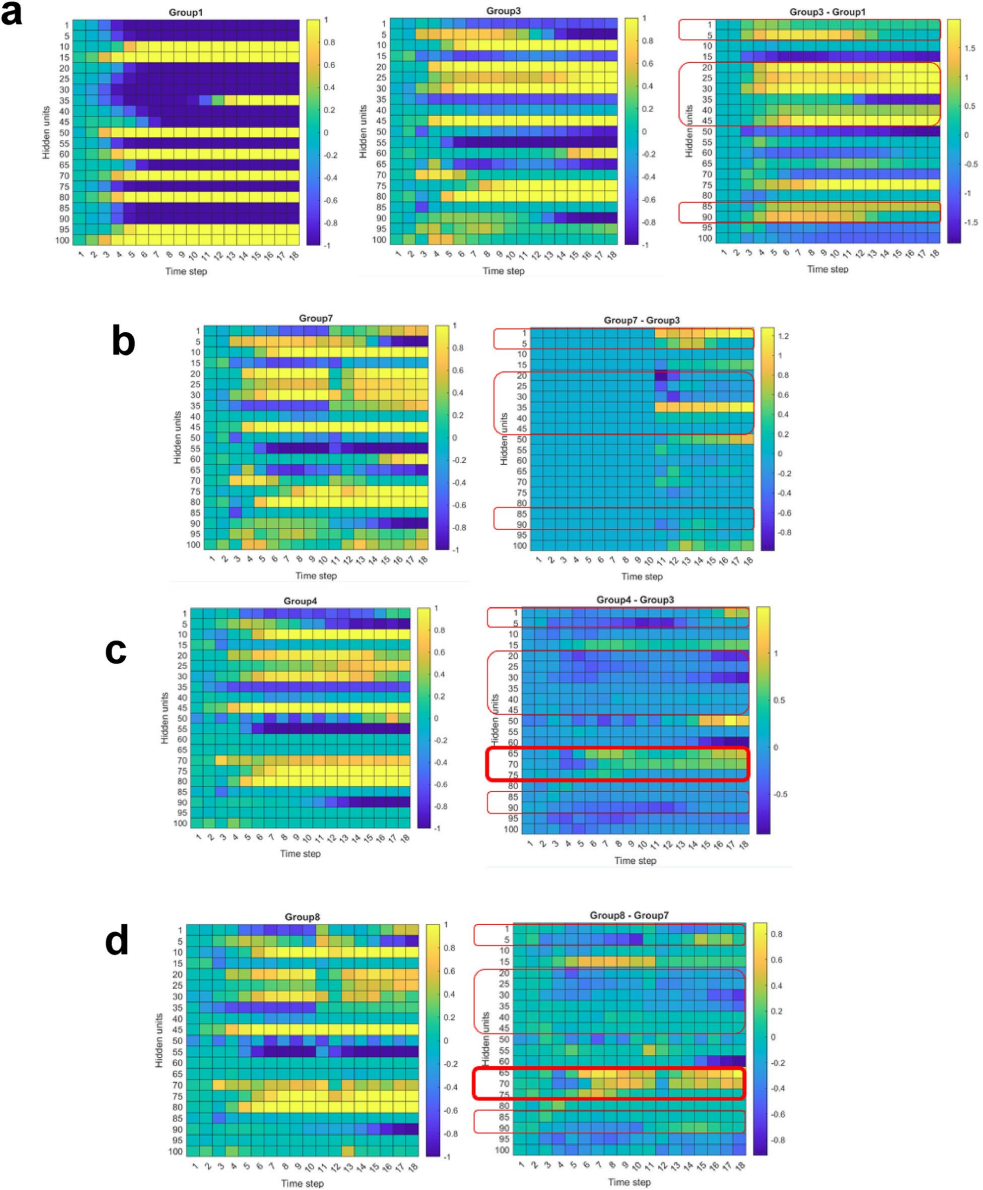
Analyses of the maps of hidden units inside the LSTM-RNN model, which has 100 hidden units at each step and 18 steps in total. At each step, the tumor volume would be a linear combination of the values of 100 hidden units. The first time step on the horizontal axis corresponds to the day when the first anti-PD-L1 was administered. (**a**) Group 1 (isotype control) versus group 3 (20 Gy on day 3, isotype control). (**b**) Group 7 (20 Gy on day 3, 20 Gy on day 13, isotype control) vs. group 3. (**c**) Group 4 (20 Gy on day 3, anti-PD-L1) vs. group 3. (**d**) Group 8 (20 Gy on day 3, 20 Gy on day 13, anti-PD-L1) vs. group 7. We hypothesize that the difference between two groups, reveals the unique therapeutic effect attributable to either anti-PD-L1 or radiation. Three highlighted regions (vertical index: 1–5, 20–45, 85–90) correspond to radiation, while the fourth highlighted region (vertical index: 65–75, bold boundaries) corresponds to anti-PD-L1.

To gain further insights into the “causal relationship”, we extracted the values of 100 hidden units for each of the 18 time steps and performed the arithmetic operation. For instance, we examined the difference between group 1 and group 3 (Fig. 6a), where both groups received the isotype control, but only group 3 received a single pulse of 20 Gy on the third time step. Therefore, in group 1, the temporal evolution of the map of hidden units primarily reflects the process of tumor growth, while the map of group 3 captures the processes of both tumor growth and the influence of radiation. As a result, the difference map between the two groups, denoted as Group 3—Group 1, disentangles the interplay between tumor growth and radiation, highlighting the evolutionary dynamics of the therapeutic effect of radiation. We speculate that the presence of more positive values within the highlighted regions yields more effective tumor control (e.g., vertical index: 1–5, 20–45, 85–90, three highlighted boxes).

A similar comparison is presented in Fig. 6b. Both group 3 and group 7 received a single pulse of 20 Gy on the 3rd time step and isotype control, but only the latter received the second pulse of 20 Gy 10 days later. Analyzing the difference thus unveils the role of the second pulse showing some intriguing characteristics. First, as expected, the difference map is filled with zeros before the 10th step since both groups share identical inputs. Secondly, after the 10th time step, the pixels within the three highlighted regions display a mix of positive and negative values, albeit of smaller magnitude compared to the first pulse (group 3—group 1 in Fig. 6a). This diminished tumor control suggests that as the tumor keeps on growing after the first pulse, it becomes more difficult to treat by radiation. Due to the current limitations of our animal experiments, we are unable to investigate the temporal response beyond the 3-week mark.

The impact of anti-PD-L1 is demonstrated in Fig. 6c, by comparing group 3 (isotype control every other day) and group 4 (anti-PD-L1 every other day). Both groups received a single pulse of 20 Gy on the 3rd time step. The difference between the two reveals the effect solely attributable to anti-PD-L1, as highlighted in a new region (vertical index: 65–75). This region contains predominantly positive values and does not overlap with the three previously highlighted regions linked to radiotherapy. While the selection of regions lacks solid evidence and requires additional validation, the results are somewhat expected, considering the distinct mechanisms through which radiation and anti-PD-L1 operate. Moreover, inside this region, we see the effect begins to emerge approximately seven steps after the first pulse and continues throughout the treatment.

The role of anti-PD-L1 antibody in the presence of two 20 Gy pulses (at the 3rd and 13th time steps) presents a new pattern, between group 7 (without anti-PD-L1) and group 8 (anti-PD-L1) in Fig. 6d. A transient perturbation (13th time step) occurs in alignment with the second pulse, indicating an instantaneous immunosuppressive effect caused by radiation. One possible explanation could be that the second radiation pulse induces cell death in both immune and tumor cells, thereby diminishing the binding between PD-1 and PD-L1 as a whole. Consequently, this attenuates the efficacy of immune checkpoint blockade.

## Discussion

The LSTM-RNN model has demonstrated its unique strength in modeling the process of tumor control in combined therapy up to three weeks. It capitalizes on its inherent ability to process sequential information, which makes it well suited for achieving our objective (Fig. 2). Its recurrent computation property is also advantageous as it prevents the growth of the number of model parameters as the number of time steps increases, particularly beneficial when dealing with small datasets.

Each group in our study corresponds to a specific input configuration, consisting of radiation dose and anti-PD-L1 drug dose. In essence, the LSTM-RNN model attempts to find the mapping between inputs and outputs for all 26 groups, which resembles a challenging curve fitting task considering the intricate and unknown mechanisms of interaction involved. Two additional challenges we encountered are: (1) the presence of many zeros in the input sequence (e.g., representing days without either radiation or anti-PD-L1 treatment), and (2) the absence of tumor volume measurement on multiple days. As a matter of fact, we conducted comparative tests and found that a feed forward network (FNN) performed much less effectively than the LSTM-RNN model. We also tested a “many-to-many” structure aiming to predict responses for all time points, including those with missing measurements. However, the results of this approach are inferior compared to the “many-to-one” structure employed in this manuscript. Such disparity is probably attributed to the increased difficulty for an AI model in establishing temporal correlations when a sequence contains numerous missing data points.

Another novel of our approach is the utilization of hidden units to identify temporal correlations between radiation and anti-PD-L1 (Fig. 6). This allows us to gain insights into the “causal relationship” from a completely new perspective. However, the biological interpretations drawn from observing the dynamics of hidden units in our work should be approached with caution. The latent representations in our model, as well as selecting different regions, are very abstract and require additional evidence to support our speculations. In this regard, we would like to reemphasize that our current goal is to identify the general trend in tumor control and potential synergy in a qualitative manner, rather than focusing on quantitative accuracy. Further quantitative analysis and interpretation may need to be postponed until additional biological correlates become available.

Additionally, the LSTM-RNN model may support in-silico studies (Fig. 5c), generating essential data to address other relevant research questions in the field of combined PULSAR and ICI therapy. Several factors can be combined as inputs, such as dose per pulse, pulse structure, anti- PD-L1 dose, and relative timing. Moving forward, we will conduct a more in-depth exploration of various permutations to identify the optimum treatment strategies for maximizing the synergy between PULSAR and ICI therapy, in parallel with the design of the next phase of experiments. In our view, daily fractions are very unlikely to be the optimal choice.

Ordinary differential equation (ODE)-based mathematical models are extensively used for analyzing complex biological systems. These models include components such as tumor cells, immune effector cells, antigens, regulatory cells, antibodies, and natural killer cells^25–29^. For instance, Eftimie et al.^25^ developed models focusing on tumor cells and immune effector cells, resembling a predator–prey model. Serre et al.^29^ utilized discrete-time equations to model the synergy between immunotherapy and radiotherapy. In the future, we plan to adopt a similar approach and compare the performance between the ODE-based and AI models. The two may complement each other and enhance their interpretation. Additionally, in our current study, the AI model focuses on the mean output of each group, without accounting for inter-animal variation. A broader question arises regarding the level of personalization in combined PULSAR and immunotherapy. Eventually, it might also be possible for us to take into account different temporal responses and identify optimum treatment strategy for each animal through reinforcement learning, similar to two previous studies^30,31^.

While it has been reported that administering anti-PD-L1 concurrently with radiation provides additional benefits^1–8^, the dynamic interplay between these two treatment modalities over time remains unclear. Figures 3 and 4 suggest the existence of an equilibrium period between cancer cell proliferation and the immune response, particularly the cytotoxic activity of CD8 + T cells. This equilibrium phase occurs 2 or 3 days after the initial radiation pulse and lasts for several days, as indicated by the plateau regions in Figs. 3c,d, 4a,b. The modulation of either PD-L1 or PD-1 levels can disrupt this equilibrium. We hold the belief that a more profound comprehension of synergy can be achieved through an exploration of the PULSAR effect, which represents an immune-mediated antitumor response that could be either inhibitory or stimulatory. From a modeling standpoint, the PULSAR effect can function as a quantitative metric to help us evaluate whether the combined impact surpasses the sum of individual contributions from two treatments. The model can be based upon not only tumor volume, but also the temporal dynamics of other biomarkers related to immune response. The following three aspects elaborate on the necessity and significance of such an exploration.

The first aspect is the timing of radiation pulses. It is important to consider that when the first radiation pulse recruits circulating CD8 + T cells from the blood into the tumor microenvironment (Fig. 1a), these newly arrived cells should not coincide with the second radiation pulse. Additionally, having two radiation pulses too close together may not be optimal as it can stimulate tumor growth. A study using similar preclinical models demonstrated that tumor growth was accelerated, and survival decreased when subsequent 3-Gy doses were administered daily following ablative radiation therapy^32^. The study also showed that repeated daily doses of 3 Gy abrogated CD8 + T-cell infiltration into tumors, which is an important predictor of immunotherapy response^32,33^.

The second aspect is related to the TME and the dynamics of T cells. The infiltration of T cells into the tumor microenvironment also plays a critical role (Fig. 1c). In “hot” tumors, there is a robust immune response characterized by significant immune cell infiltration and activation of immune pathways. Conversely, “cold” tumors exhibit limited immune response, with minimal immune cell infiltration and reduced immune activity. Our previous study already found that the “cold” LLC tumor model responded best to radiation doses spaced 10 days apart, with only the second PD-L1 dose required for maximizing therapeutic effect. On the other hand, in “hot” tumor models such as mouse colon carcinoma (MC38), a single radiation dose is optimal, as initial priming of the immune response has already occurred due to preexisting tumor immunity^11,34,35^. There is a need for an investigation into how T cell infiltration is temporally influenced by radiation pulses concerning dose and spacing. Understanding this behavior holds significant implications for treatment strategies, enabling PULSAR to effectively target the specific immune characteristics of different tumor types. Moreover, the impact of PULSAR on other leukocytes, such as neutrophils and macrophages, remains unknown, despite their demonstrated importance in preclinical models for a-PD-L1 therapy response^36–38^. Furthermore, a study reported that intra-tumoral T cells, particularly T_reg_ cells, can mediate tumor control without the influx of newly infiltrating T cells and exhibit greater resistance to radiation compared to T cells in other body compartments, like memory T cells^19^. This suggests that if repeated radiation were to longitudinally amplify the regulatory effects of T_reg_ cells, the impact of PD-L1 antibody might be intensified, and as a result, anti-PD-L1 administration should be correspondingly enhanced.

The third aspect involves T_reg_ cells. T_reg_ cells, known for their role in regulating anti-tumor immunity both inside and outside the TME, are believed to be a major contributor to immune escape mechanisms in cancer. In one study, the anti-CD25 antibody in a murine prostate tumor model significantly enhanced the efficacy of radiation, resulting in delayed tumor growth^15^. In a murine mesothelioma model, CTLA4 blockade reduced the proportion of T_reg_ cells relative to effector T cells after radiation, boosting the activation of CD8 + T cells^17^. In another study in which mice were irradiated with 10 Gy gamma radiation (prostate C1 cells)^16^, T_reg_ cells significantly increased in the spleen, lymph nodes, blood, and lung within 2 days after exposure, returning to normal levels by 10 days. The authors also reported three dose-dependent patterns: (1) T_reg_ cells increase even after a low dose of 2 Gy, (2) mice receiving over 10 Gy irradiation exhibited double the fraction of splenic T_reg_ cells compared to the control group, although there was no significant difference between 10 and 20 Gy, and (3) fractionating a larger dose into smaller daily fractions (e.g., 10 Gy vs. 5 × 2 Gy, 20 Gy vs. 3 × 8 Gy) slightly reduced the production of T_reg_ cells. Comparable analyses can be incorporated with PULSAR and our suggested AI modeling framework to further investigate the synergy and “causal relationship”.

In this study, our focus was on developing an AI model to study the synergy between radiotherapy and ICI therapy. To the best of our knowledge, this is the first work of its type. Nevertheless, the tasks presented in this manuscript constitute only the very first phase, with several limitations requiring further exploration. Firstly, the large error bars depicted in Figs. 3 and 4 suggest significant variability in measured tumor volumes within each group, stemming from varying responses of animals and human errors. Since our goal is to discern overall trends, the AI model concentrates on the mean output of each group. Data augmentation was utilized to produce additional training samples, intentionally reducing the standard deviation by a factor of five. In future investigations, we plan to broaden the evaluation by assessing how the model’s performance varies with different scaling factors. As more datasets become available, we will explore the robustness and generation capability of AI models, like the analysis presented in Figure S2 in the supplemental materials. Moreover, for enhanced precision in volume measurements, upcoming studies should contemplate the adoption of advanced contouring techniques based on CBCT and PET images.

Secondly, selecting tumor volume as the sole output metric limits the biological insights, lacking data on immune cell infiltration and functions. To address that limitation, we plan to perform additional experiments utilizing immunohistochemistry or flow cytometry to provide insights into the temporal behavior of CD8 + T cells, T_reg_ cells, and myeloid cells^39^. The sequential measurements of these biomarkers will be conducted. Intra-vital optical imaging tools can also be employed to study how T cells migrate from lymph nodes to the tumor via the circulatory system, and subsequently infiltrate the tumor^40,41^. Given the inherent flexibility of the AI model, it would be a straightforward process to integrate this additional biological information. In addition, a more continuous measurement of tumor volume would be advantageous. The preferred choice is to use the daily tumor volume as the output instead of the tumor volume change, as indicated in this manuscript (Eq. 1).

Thirdly, the limited number of groups (26 in total) does not provide sufficient data diversity. Our plans include expanding the dataset by incorporating more combinations and conducting additional animal experiments. It is crucial for us to validate the AI models using different cell types and tumor models, as each is expected to demonstrate unique synergies. Furthermore, additional investigations are necessary to understand how the findings in mouse models translate to clinical settings^42,43^. Mouse tumors grow notably faster than human tumors and are more radioresistant. Considering the longer half-life of PD-L1 antibodies in the human body (e.g., > 30 days), it is speculated that a longer interval between radiation pulses in clinical PULSAR may be necessary compared to preclinical models.

## Conclusions

We have successfully demonstrated that the proposed LSTM-RNN model exhibits unique strengths in predicting tumor growth and identifying potential “causal relationships”, based on the results of preclinical experiments. Our study introduces a novel perspective on studying the temporal interaction between radiation and ICI therapy. When used as an in-silico modeling tool, the LSTM-RNN model can maximize therapeutic effects through the optimization of variables such as pulse interval, radiation dose, drug dose, and timing. By implementing more rigorous experiments and dedicating future efforts to biomarker identification, we can deepen our comprehension of biological mechanisms and improve quantitative capabilities.

### Supplementary Information

Supplementary Information.

## Data Availability

Data that support the findings of this study are available from the corresponding author upon request.
