# Supplementary material for: An AI-based approach for modeling the synergy between radiotherapy and immunotherapy
Source: Sci Rep. 2024 Apr 8;14:8250. doi: 10.1038/s41598-024-58684-6 (PMC11001871; doi:10.1038/s41598-024-58684-6)
Supplement: Supplementary file 1 — Supplementary Information. [file 41598_2024_58684_MOESM1_ESM.docx]

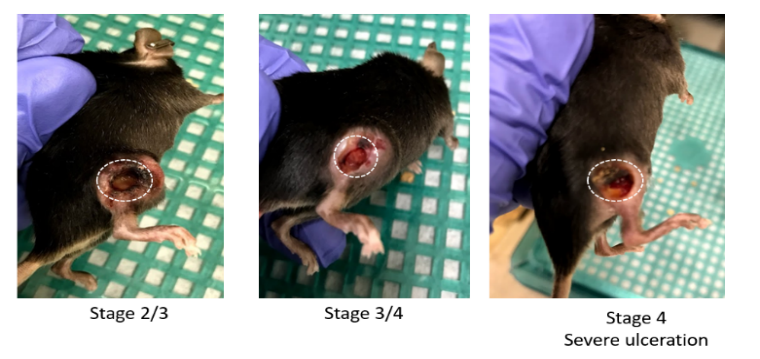

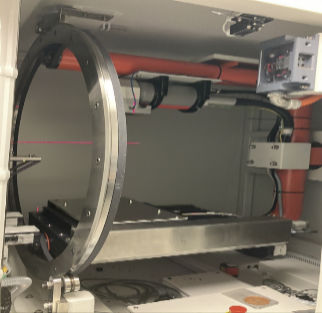


**Fig. S1.** (**left**) Experiential set up for radiation treatment. Tumor-bearing mice were irradiated with 10 to 40 Gy according to multiple schedules (**Table S1**), using local irradiations with a small animal dedicated x-ray irradiator (X-RAD 32, Precision X-ray, Inc. (**right**) The tumor volumes were measured by length (x), width (y), and height (z) and calculated as xyz/2. If the tumor volume exceeds 1500 mm^3^, or the mouse has significant ulceration in the tumor, the mouse reaches the survival endpoint and is euthanized.

**Table S1.** The timing diagram of the combined therapy in our study (26 groups in total). For immunotherapy, either anti-PD-L1 or isotype control was administered. For radiation, different doses were delivered (10, 15, 20, 40 Gy). The first pulse of radiation was delivered 14 days after the implantation. Tumor volume measurements were carried out sequentially on certain days.


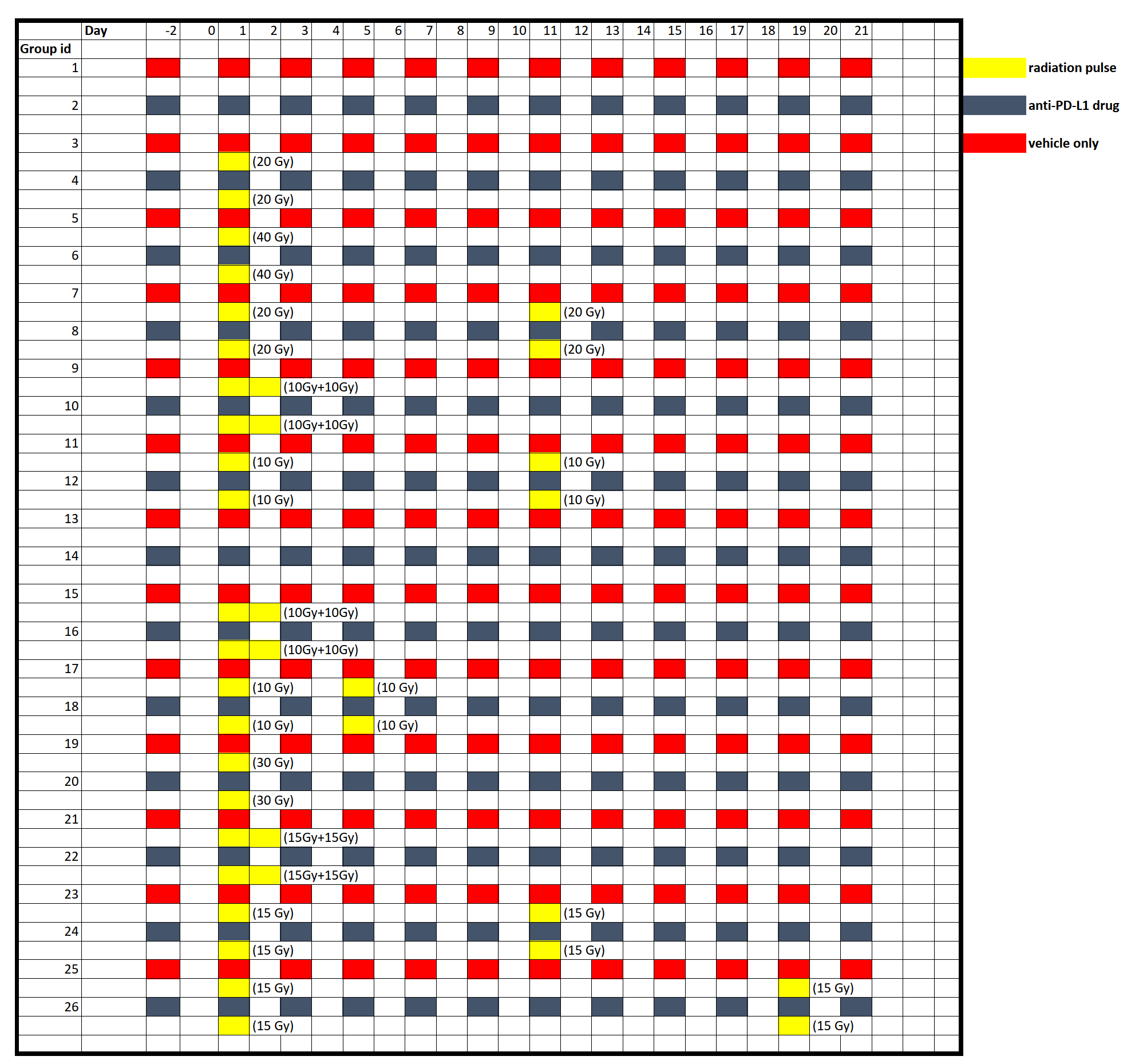


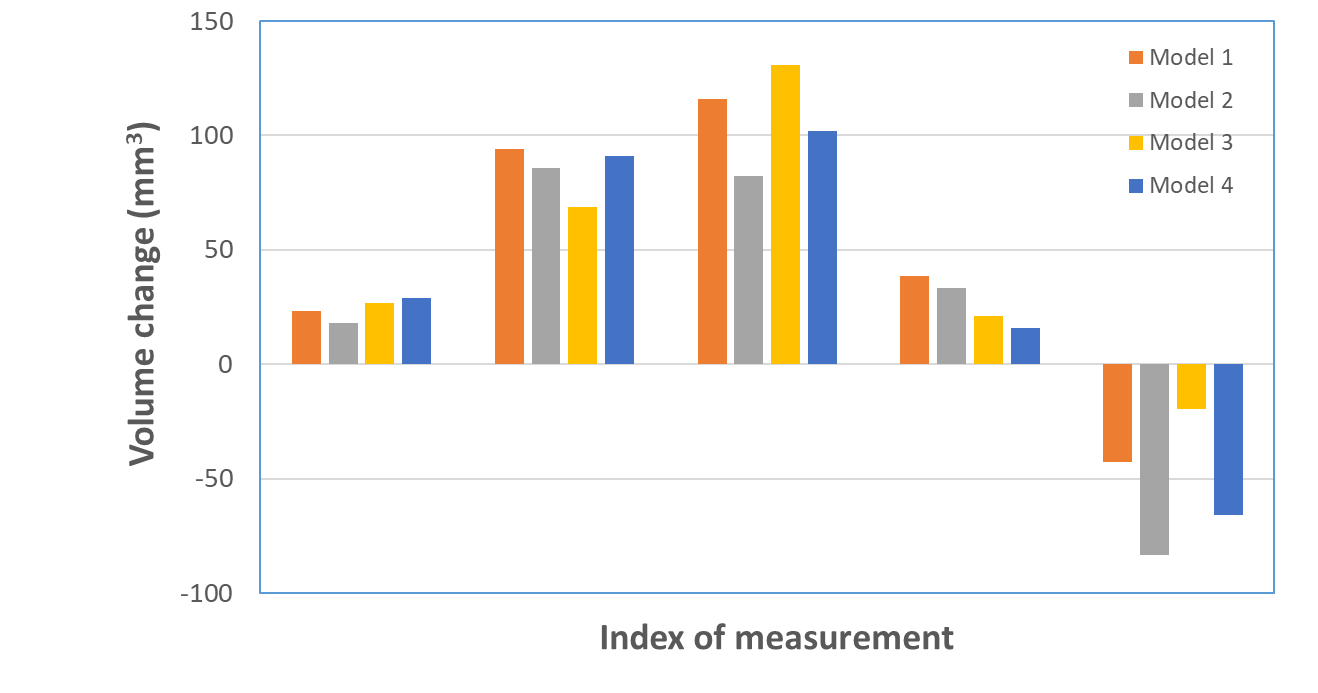


**Fig. S2.** Illustration of performance difference among four models for a new treatment scheme. To train a model, two groups out of 26 were randomly selected and excluded from the training dataset. The result suggests the limitation due to the small dataset in our current study.

**
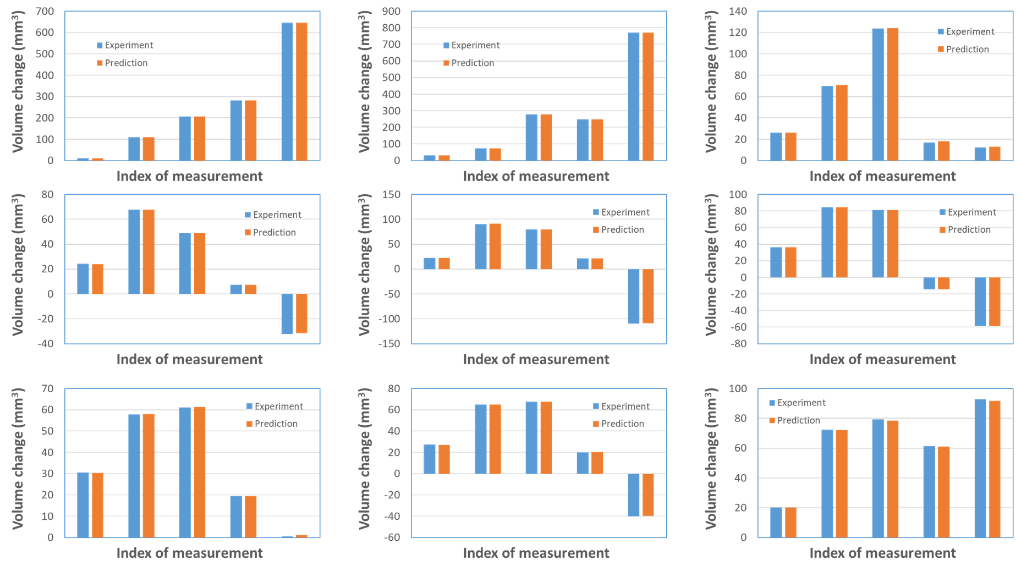
**

**
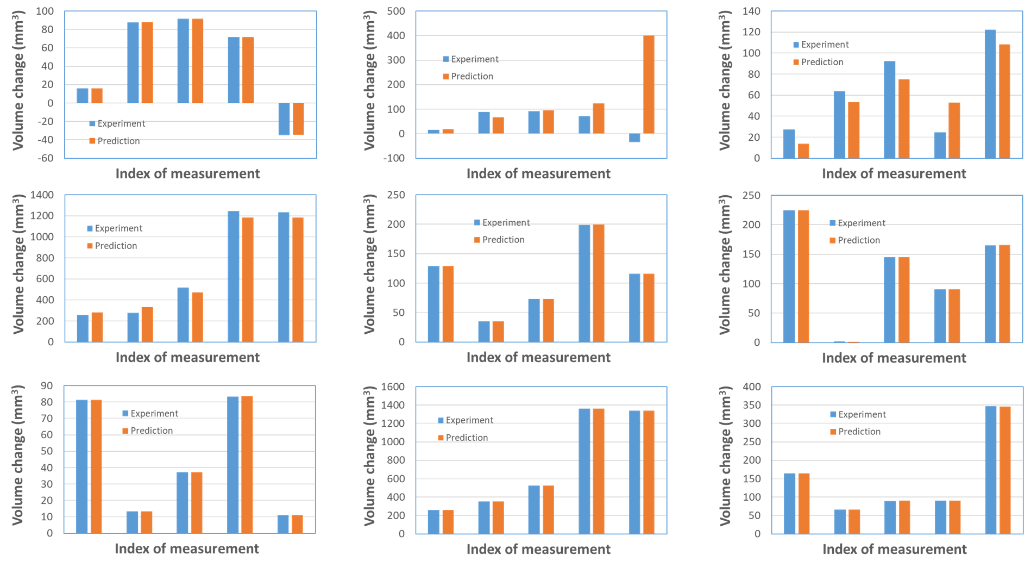
**

**
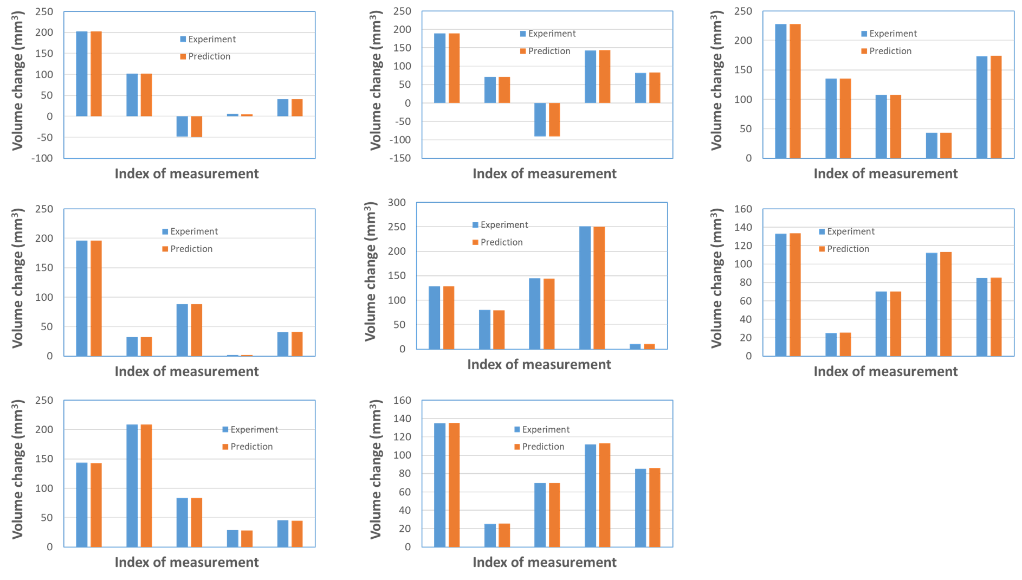
**

**Fig. S3.** Predicted tumor volume change for all 26 groups in comparison with the results of five measurements based on one selected LSTM-RNN model.
